# Oxylipin Dynamics Following A Single Bout of Yoga Exercise: A Pilot Randomized Controlled Trial Secondary Analysis

**DOI:** 10.1089/jicm.2024.0233

**Published:** 2024-09-16

**Authors:** Dennis Muñoz-Vergara, Pamela M. Rist, EunMee Yang, Gloria Y. Yeh, Norman Lee, Peter M. Wayne

**Affiliations:** ^1^ Osher Center for Integrative Health, Brigham and Women’s Hospital, Harvard Medical School, Boston, MA, USA.; ^2^ Division of Preventive Medicine, Brigham and Women’s Hospital, Harvard Medical School, Boston, MA, USA.; ^3^ Division of General Medicine and Primary Care, Beth Israel Deaconess Medical Center, Harvard Medical School, Boston, MA, USA.; ^4^ Metabolite Profiling Core Facility, Koch Institute for Integrative Cancer Research, Massachusetts Institute of Technology, Cambridge, MA, USA.

**Keywords:** Mind-body movement therapies, yoga, exercise, inflammatory lipid mediators, oxylipins

## Abstract

**Background:**

Yoga may promote health via a complex modulation of inflammation. Little is known about oxylipins, a class of circulating mediators involved in inflammation resolution.

**Objective:**

To explore the acute effects of yoga exercise on systemic levels of oxylipins.

**Methods:**

This is a secondary analysis of a three-arm (high-intensity-yoga: HY, *n* = 10); moderate-intensity-yoga: MY, *n* = 10; and no-intervention-control: CON, *n* = 10) pilot randomized controlled trial employing a single bout of yoga exercise. Blood samples (baseline and 4-timepoint post-intervention) were used for an unbiased metabolipidomic profiling analysis. Net Areas Under the Curve per oxylipin were evaluated for each group.

**Results:**

Lipoxin(LX)B4, prostaglandin(PG)D2, and resolvin(Rv)D3 exhibited a greater magnitude of change in HY compared with MY and CON.

**Conclusion:**

Findings inform the design of future trials exploring the acute effects of yoga exercise on oxylipins’ systemic levels.

## Introduction

Mind-body movement therapies (MBMT), such as yoga (a multi-component mind-body practice that originated in India/South Asia), are thought to afford health benefits by modulating multiple body systems.^1^ The immune system and its related inflammatory processes are generally appreciated in the context of infections and the development of chronic health conditions.^2^ However, research shows they also play physiological roles, such as tissue regeneration, remodeling, and stress adaptation.^3^ Exercise is one example of physiological inflammation modulated by the intensity, duration, and frequency of practice.^4^ Previous studies have demonstrated that a single bout of physical activity (PA) can induce systemic changes in cytokines and immune cells, as well as overall adaptation of these processes after long-term training.^5–7^ A recent meta-analysis suggests that MBMT may also induce health effects via changes in systemic levels of cytokines.^8^ However, little is known about the circulating dynamics of another group of inflammatory mediators after PA or MBMT, collectively known as oxylipins.^9,10^ Oxylipins are a group of bioactive lipid mediators in charge of orchestrating inflammation initiation and active resolution.^11^

This study aims to evaluate the impact of a single bout of yoga exercise (i.e., āsana) on the systemic levels of a set of oxylipins. This secondary analysis emanated from a recently published pilot RCT that characterized the changes in systemic levels of cytokines to contribute to the scarce MBMT scientific literature on the systemic physiological effects of yoga exercise.^12^

## Material and Methods

### Study design

These results emerged from secondary analyses of a three-arm, pilot randomized controlled trial (RCT) of a single bout of yoga exercise intervention. In brief, the RCT included three acute exposures (30 participants total): high-intensity (HY) and moderate-intensity yoga exercise (MY) groups and a no-intervention control (CON). Details of the study design and outcomes related to study feasibility and preliminary cytokine findings are described elsewhere.^12^

#### Yoga exercise groups

A certified Hatha yoga instructor (DMV) administered individual, one-on-one yoga sessions. The protocol for both groups consisted of the same sequence of postures and was delivered over 60 min (Supplementary Table S1 and S2). During the intervention, participants were offered feedback, specific cues, and posture modifications. No explicit instructions in other yoga components were provided (e.g., imagery).

#### Control group

Participants rested in a quiet room in the same clinical facility for one h and were asked not to engage in physical, mental, or emotional activity.

### Randomization and blinding

An allocation sequence (i.e., random permuted blocks of sizes 3 and 6) was generated and uploaded to the Research Electronic Data Capture (REDCap) online system to randomize participants. Participants and instructor were not blinded. Nurses and laboratory technicians were blinded during sample collection and laboratory analyses. The data analyst was unblinded after completing all laboratory analyses.

### Sample collection during study visits

Baseline blood draws were taken, followed by randomization. At the end of the intervention, nurses performed serial blood sampling (0-, 60-, 120-, and 180-min). Samples were immediately centrifuged to obtain plasma, aliquoted, and stored at −80°C.

#### Unbiased metabolipidomic profiling

Baseline, 0-, 60-, 120-, and 180-min samples were analyzed: In a 1.5 Eppendorf tube, 100 µL of plasma was mixed with an organic solution (Acetonitrile:Methanol:Acetone—8:1:1) at a ratio of 1:8 and vortexed for mixing. Samples were incubated at 4°C/30-min to allow proteins to precipitate. After spinning samples at 15,000 RCF/15-min/4°C, supernatants were transferred to clean tubes and placed in a speed vacuum centrifuge at 35°C/2-h. Pellets were reconstituted with a 1:1 Methanol:H20 ultrapure solution and vortexed until completely dissolved. Supernatants were transferred to Liquid Chromatography Mass Spectrometry (LC-MS) vials with insert (ALWSCI Technologies Co.). Liquid chromatography–tandem mass spectrometry measured lipid mediators using a Sciex API4000 (Sciex) with Agilent 1200 LC and a PAL autosampler. Buffer A: ultrapure water + 0,1% formic acid. Buffer B: acetonitrile + 0.1% formic acid. The flow rate was 0.2 mL/min, and each run was 5% to 95% of buffer B for 20 min. Identification was conducted with signature ion fragments for each target lipid mediator using a minimum of six diagnostic ions. Quantification was achieved using calibration curves. Internal standards (Cayman Chemicals) used to quantify levels of 20 lipid mediators: SPM D-series LC-MS Mixture; SPM E-series MaxSpec^®^ LC-MS Mixture; Lipoxin MaxSpec^®^ LC-MS Mixture; Primary COX and LOX MaxSpec^®^ LC-MS Mixture. Examples of oxylipins and their purported role in inflammation are listed in Supplementary Table S3.

### Statistical methods

Data were analyzed and plotted with descriptive statistics (GraphPad Software, Inc., San Diego, CA, USA). Continuous raw data were tested for normality using the Shapiro–Wilk Test and Q-Q plots. Means and standard error of the mean were reported for normally distributed data. Otherwise, medians and interquartile ranges for non-normally distributed data.

Undetectable oxylipin levels at a given timepoint were not included in analyses and were treated as truncated data. To characterize oxylipin responses, data from each timepoint were used to calculate net Areas Under the Curve (netAUC) adjusted for each participant’s baseline value.^12^ This analysis compares the increases and decreases of each oxylipin over time using the trapezoid rule to summarize the change in each oxylipin level as a single value. These netAUC values were also used to calculate Cohen’s d effect sizes (i.e., small = 0.2; medium = 0.5; large = 0.8). Given the pilot nature of this study and sample size, these analyses were exploratory. The sample size for this study was expected to be sufficient for evaluating *a priori*-defined feasibility metrics. Data were reported and analyzed according to the intention-to-treat principle.

## Results

Participants’ baseline characteristics are summarized elsewhere (Supplementary Table S4).^12^

###  

#### Systemic levels of oxylipins before and after yoga exercise

**FIG. 1.**
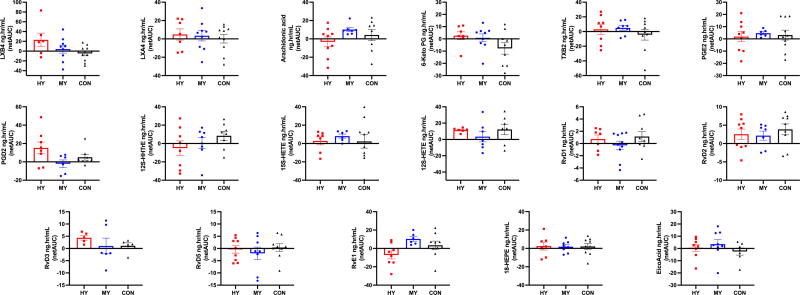
Bar graphs denote each group’s net Area Under the Curve (netAUC) mean and standard error of the mean (SEM) per oxylipin. Timepoints: baseline, 0-, 30-, 60-, 120-, and 180-min were included in the netAUC calculation. High-Intensity yoga exercise group netAUC in squares (HY); moderate-intensity yoga exercise group netAUC in circles (MY); control group netAUC in triangles (CON).

Levels of 17 oxylipins and three precursors were measured. Figure 1 synthesizes the effects of yoga exercise on oxylipin levels using netAUC analyses. For prostaglandin (PG) F2, 5-Hydroxyeicosatetraenoic Acid (5S-HETE), and Docosahexaenoic acid (DHA), incomplete data precluded this analysis. Descriptive comparisons between groups of netAUC scatter plots revealed three main patterns: (1) for three oxylipins (LXB4, PGD2, and RvD3), the magnitude of netAUC change for the HY group was greater than for the MY and CON groups. This indicates that levels of these oxylipins increased more, relative to baseline, in the HY group; (2) in most cases, the magnitude of changes between the two yoga groups did not show any consistent response, except for AA and RvE1 with an increased in MY versus a decreased in HY; (3) for the other oxylipins, no clear differences between the groups were observed. Cohen’s d effect sizes were calculated between groups’ netAUC. In yoga groups, four oxylipins showed large effect sizes: RvE1 (HY = 0.8); LXB4 (HY = 1.1), 6-keto-PGF1α, (HY = 0.9), and PGD2 (MY = 0.9) (Supplementary Table S5).

## Discussion

The study of physiological processes associated with practices that explicitly engage the myofascial system is still in its early stages (e.g., MBMT). This MBMT pilot RCT quantified acute changes in circulating levels of pro-inflammatory and pro-resolution oxylipins. The study successfully collected and analyzed plasma samples of 17 DHA- and EPA-derived omega-3 and AA-derived omega-6 oxylipins and identified candidates such as PGD2, LXB4, and RvD3, to be researched in future studies.

Despite the exploratory nature of this study, these analyses contribute novel findings characterizing changes in oxylipins following a single bout of yoga exercise and extend previous research evaluating the impact of conventional PA on oxylipins to MBMT.^9^ Specifically, three oxylipins (i.e., PGD2, LXB4, and RvD3) were identified to be particularly responsive to the HY intervention. These oxylipins show anti-inflammatory and pro-resolving roles; however, their roles in the myofascial system during MBMT practice have yet to be explored.^13,14^ PA studies have reported increases in oxylipins (e.g., TXB2, PGE2, and PGF2α) immediately after short-term resistance and endurance exercise interventions.^9,15^

Of note, this study does not address the longer-term effects of MBMT on systemic levels of oxylipins. The scientific literature indicates that long-term exercise interventions involve an intricate physiological adaptation process. Within a few hours of the PA practice, cytokines and oxylipins orchestrate the initiation and resolution of inflammatory processes in the myofascial system.^5,9^ Once the PA intervention is repeated over time, a physiological adjustment occurs, dampening the basal levels of these molecules, especially those with a pro-inflammatory profile.^6,9^ In this context, exercise has been experimentally used to understand the temporal dynamic of circulating bioactive mediators resulting from a self-regulated inflammatory process at the myofascial system. Future fully powered studies will be needed to further examine how different types of conventional PA versus MBMT, as well as intervention parameters, such as intensity, frequency, and duration, impact oxylipin systemic dynamics.^4^

### Strengths and limitations

This study contributes to the limited scientific literature studying the physiological effect of MBMT on bioactive inflammatory mediators.^8,12^ Of note, this is the first RCT to explore the dynamic of circulatory oxylipin levels after a single bout of yoga exercise. In addition, it was utilized a validated metabolomics-based methodology, targeting omega-3 and –6-derived oxylipins involved in initiating and resolving inflammation.^16^

This exploratory analysis has limitations. The study included yoga-naïve and inactive adults, which does not allow generalizability to other groups (e.g., children). The randomization process did not prevent imbalances due to the small sample size. There is a risk of bias because the instructor and participants were aware of the assigned intervention during the trial. Additional sources of heterogeneity include the top-down neuro-muscular influences and the potentially confounding added element of mental engagement (e.g., focused attention), which was not isolated from the physical component. Owing to the small sample size, this pilot study was not powered to formally evaluate group differences. Hence, future fully-powered studies are needed for these purposes.

## Conclusions

It is feasible to characterize oxylipin dynamics following a single bout of yoga exercise. Future MBMT studies evaluating systemic dynamics of key oxylipins in the context of physiological inflammatory processes warrant further research.

## Data Availability

This article and its supplementary information files include the datasets used and/or analyzed during the current study. The raw datasets are available from the corresponding author on reasonable request.
