# Supplementary material for: Oxylipin Dynamics Following A Single Bout of Yoga Exercise: A Pilot Randomized Controlled Trial Secondary Analysis
Source: J Integr Complement Med. 2024 Sep 16;30(9):897–901. doi: 10.1089/jicm.2024.0233 (PMC11807855; doi:10.1089/jicm.2024.0233)
Supplement: Supplementary Data [file jicm.2024.0233_suppl_data.pdf]

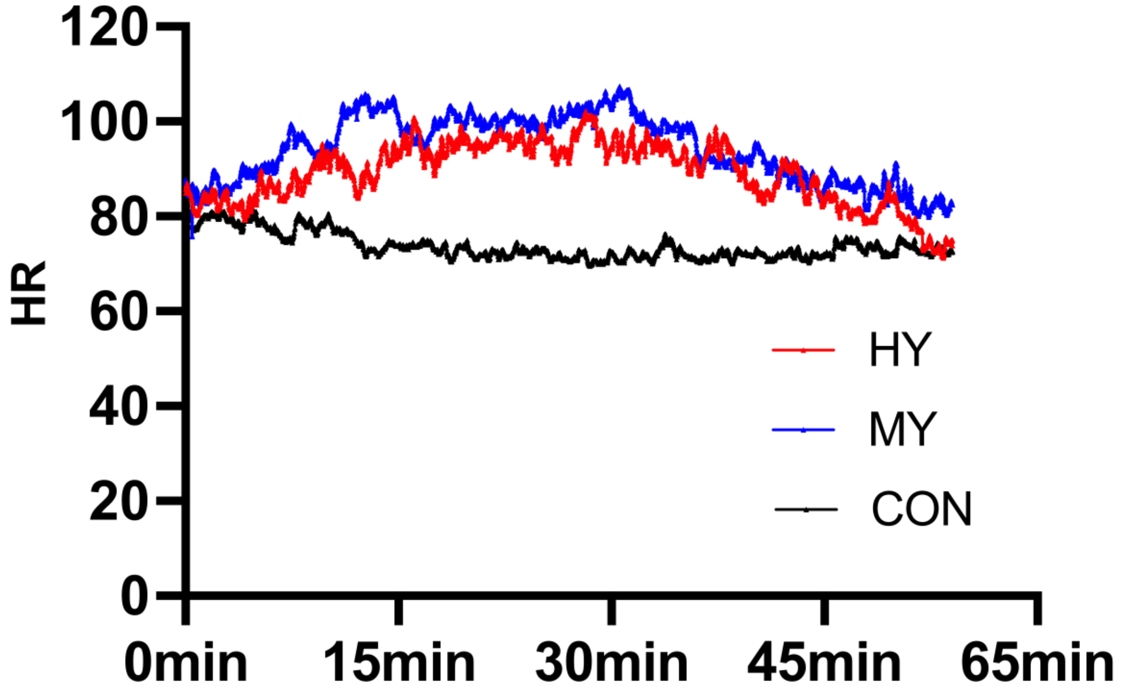


Measure of heart rate (HR) to characterize changes in each group. One-hour longitudinal trajectories of HR for each group. High-intensity yoga exercise group (HY), moderate-intensity yoga exercise group (MY), and control group (CON).

REFERENCE LIST

This reference list provides the Inflammatory and myofascial roles of Omega-6 (AA-derived oxylipins) and Omega-3 (DHA- and EPA-derived oxylipins) derived lipid mediators.


1. Dyall SC, Balas L, Bazan NG, et al. Polyunsaturated fatty acids and fatty acid-derived lipid mediators: Recent advances in the understanding of their biosynthesis, structures, and functions. Prog Lipid Res 2022;86(101165, doi:10.1016/j.plipres.2022.101165
2. Feehan KT, Gilroy DW. Is Resolution the End of Inflammation? Trends Mol Med 2019;25(3):198-214, doi:10.1016/j.molmed.2019.01.006
3. Gangemi S, Luciotti G, D'Urbano E, et al. Physical exercise increases urinary excretion of lipoxin A4 and related compounds. J Appl Physiol (1985) 2003;94(6):2237-40, doi:10.1152/japplphysiol.01004.2002
4. Vella L, Markworth JF, Farnfield MM, et al. Intramuscular inflammatory and resolving lipid profile responses to an acute bout of resistance exercise in men. Physiol Rep 2019;7(13):e14108, doi:10.14814/phy2.14108
5. Dorris SL, Peebles RS, Jr. PGI2 as a regulator of inflammatory diseases. Mediators Inflamm 2012;2012(926968, doi:10.1155/2012/926968
6. Markworth JF, Vella L, Lingard BS, et al. Human inflammatory and resolving lipid mediator responses to resistance exercise and ibuprofen treatment. Am J Physiol Regul Integr Comp Physiol 2013;305(11):R1281-96, doi:10.1152/ajpregu.00128.2013
7. Duchesne E, Tremblay M-H, Côté CH. Mast cell tryptase stimulates myoblast proliferation; a mechanism relying on protease-activated receptor-2 and cyclooxygenase-2. BMC Musculoskeletal Disorders 2011;12(1), doi:10.1186/1471-2474-12-235
8. Velica P, Khanim FL, Bunce CM. Prostaglandin D2 inhibits C2C12 myogenesis. Mol Cell Endocrinol 2010;319(1-2):71-8, doi:10.1016/j.mce.2010.01.023
9. Markworth JF, Maddipati KR, Cameron-Smith D. Emerging roles of pro-resolving lipid mediators in immunological and adaptive responses to exercise-induced muscle injury. Exerc Immunol Rev 2016;22(110-34
10. Yamaguchi A, Botta E, Holinstat M. Eicosanoids in inflammation in the blood and the vessel. Front Pharmacol 2022;13(997403, doi:10.3389/fphar.2022.997403
11. Kulkarni A, Nadler JL, Mirmira RG, et al. Regulation of Tissue Inflammation by 12-Lipoxygenases. Biomolecules 2021;11(5), doi:10.3390/biom11050717
12. Giannakis N, Sansbury BE, Patsalos A, et al. Dynamic changes to lipid mediators support transitions among macrophage subtypes during muscle regeneration. Nat Immunol 2019;20(5):626-636, doi:10.1038/s41590-019-0356-7
13. Markworth JF, Brown LA, Lim E, et al. Resolvin D1 supports skeletal myofiber regeneration via actions on myeloid and muscle stem cells. JCI Insight 2020;5(18), doi:10.1172/jci.insight.137713
14. Halade GV, Dorbane A, Ingle KA, et al. Comprehensive targeted and non-targeted lipidomics analyses in failing and non-failing heart. Anal Bioanal Chem 2018;410(7):1965-1976, doi:10.1007/s00216-018-0863-7
