# Supplementary material for: Oxylipin Dynamics Following A Single Bout of Yoga Exercise: A Pilot Randomized Controlled Trial Secondary Analysis
Source: J Integr Complement Med. 2024 Sep 16;30(9):897–901. doi: 10.1089/jicm.2024.0233 (PMC11807855; doi:10.1089/jicm.2024.0233)
Supplement: Supplementary Table S1 [file jicm.2024.0233_suppl_tables1.pdf]

S1 Table. List of postures used during the yoga exercise intervention.

Sanskrit name	English name	Modification	Floor postures	Standing postures
Mārjāryāsana / Biḍālāsana	Cat/Cow	Seated version	X	
Tāḍāsana	Mountain pose (static)			X
Ūrdhva Vṛkṣāsana	Hands up			X
Uttānāsana A	Folding fold	Unlocked knees		X
Uttānāsana B	Lengthen spine			X
Kumbhakāsana	Plank	Knees on floor		X
Caturaṅga Daṇḍāsana	Halfway down	Knees on floor	X	
Urdhva Mukha Śvānāsana	Upward facing dog	Baby cobra	X	
Adho Mukha Śvānāsana	Downward facing dog	Unlocked knees / hills up		X
Samasthiti	Hands next to hips (static)			X
Vīrabhadrāsana I	Warrior I	Wall supported		X
Vīrabhadrāsana II	Warrior II	Wall supported		X
Utthita Trikoṇāsana	Extended triangle	Unlocked knees		X
Prasārita Pādottānāsana	Wide legged forward fold	Unlocked knees		X
Skandāsana	Side lunge	Wall supported		X
Vṛkṣāsana	Tree pose	Wall supported		X
Utkaṭāsana	Chair pose	Wall supported		X
Ardha Candrāsana	Half moon			X
Bālāsana	Child pose	Block supported	X	
Kapotāsana	Pigeon pose	Supine version	X	
Parivṛtta Pārśva Upaviṣṭa Konāsana	Revolved seated angle	Wall supported	X	
Paścimottānāsana	Seated forward bend or fold	Unlocked knees	X	
Setu Bandhāsana	Bridge pose	Half bridge version	X	
Ardha Matsyendrāsana	Seated spinal twist		X	
Supta Matsyendrāsana	Supine Spinal twist		X	
Jaṭhara Parivartanāsana	Knee down twist		X	
Śavāsana	Corpse pose (static)		X	
