# Supplementary material for: Oxylipin Dynamics Following A Single Bout of Yoga Exercise: A Pilot Randomized Controlled Trial Secondary Analysis
Source: J Integr Complement Med. 2024 Sep 16;30(9):897–901. doi: 10.1089/jicm.2024.0233 (PMC11807855; doi:10.1089/jicm.2024.0233)
Supplement: Supplementary Table S2 [file jicm.2024.0233_suppl_tables2.pdf]

S2 Table. List of 10 targeted anatomical groups during the yoga exercise session.

Anatomical groups
Hip extensor & adductors stretching
Hip & Plantar flexors stretching
Shoulder extensors stretching
Shoulder horizontal adductor stretching
Shoulder extensors-adductors stretching
Wrist flexor stretching
Trunk extensors stretching
Lateral flexors stretching
Trunk rotators stretching
Split stretching
