# Supplementary material for: Oxylipin Dynamics Following A Single Bout of Yoga Exercise: A Pilot Randomized Controlled Trial Secondary Analysis
Source: J Integr Complement Med. 2024 Sep 16;30(9):897–901. doi: 10.1089/jicm.2024.0233 (PMC11807855; doi:10.1089/jicm.2024.0233)
Supplement: Supplementary Table S3 [file jicm.2024.0233_suppl_tables3.pdf]

S3 Table. Inflammatory and myofascial roles of Omega-6 (AA-derived oxylipins) and Omega-3 (DHA- and EPA-derived oxylipins) derived lipid mediators.

Group	Abbreviation	Inflammatory role	Myofascial role	Reference
	Omega-6 oxylipins			
AA-derived oxylipins	AA	Precursor of Omega-6 oxylipins		1
	LXA4	Anti-inflammatory/Pro-resolving	Unknown	1,2
	LXB4	Anti-inflammatory/Pro-resolving	Unknown	3
	6-keto PGF1α	Anti-inflammatory PGI2 subrogate	Unknown	4 5
	TXB2	Pro-inflammatory TXA2 subrogate	Unknown	6
	PGE2	Pleotropic (pro- and anti-inflammatory functions)	↑ Myoblast proliferation<br>↑ Myoblast differentiation<br>↓ Myoblast fusion<br>↑ Protein degradation	7,8
	PGD2	Anti-inflammatory/Pro-resolving	↑ Myoblast proliferation<br>↓ Myoblast differentiation	7,8
	PGF2α	Pro-inflammatory	↑ Myoblast number<br>↑ Myoblast fusion<br>↑ Protein synthesis<br>↑ Myotube hypertrophy	9
	12-HHTrE	Pro-inflammatory TXA2 subrogate	Unknown	4
	15-HETE	Pleotropic (pro- and anti-inflammatory functions)	Unknown	10
	12-HETE	Pro-inflammatory	Unknown	11
	5-HETE	Anti-inflammatory	Unknown	10
	Omega-3 oxylipins			
EPA-derived oxylipins	EPA	Precursor of E series resolvins		1
	RvE1	Anti-inflammatory/Pro-resolving	Myotubes signaling regulation	12
	18-HEPE	Anti-inflammatory/Pro-resolving	Unknown	1
DHA-derived oxylipins	DHA	Precursor of D series resolvins		1
	RvD1	Anti-inflammatory/Pro-resolving	↑ Myofiber regeneration<br>Action on muscle stem cells	1,13
	RvD2	Anti-inflammatory/Pro-resolving	↑ Muscle regeneration	12
	RvD3	Anti-inflammatory/Pro-resolving	Unknown	1,14
	RvD5	Anti-inflammatory/Pro-resolving	Unknown	1,14

Abbreviations: AA, Arachidonic Acid; LX, Lipoxin; PG, Prostaglandin; TX, Thromboxane; 12-HHTrE, 12-Hydroxyheptadecatrienoic Acid; HETE, Hydroxyeicosatetraenoic Acid; EPA, Eicosapentaenoic Acid; Rv, Resolvin; 18-HEPE, Hydroxyicosapentaenoic Acid; DHA, Docosahexaenoic Acid
