# Supplementary material for: Oxylipin Dynamics Following A Single Bout of Yoga Exercise: A Pilot Randomized Controlled Trial Secondary Analysis
Source: J Integr Complement Med. 2024 Sep 16;30(9):897–901. doi: 10.1089/jicm.2024.0233 (PMC11807855; doi:10.1089/jicm.2024.0233)
Supplement: Supplementary Table S4 [file jicm.2024.0233_suppl_tables4.pdf]

S4 Table. Demographic characteristics of the study participants by randomized treatment assignment.

	Randomized groups		
	Mean ± SD, n(%)		
Variable	High-intensity yoga exercise group (HY) (n=10)	Moderate-intensity yoga exercise group (MY) (n=10)	Sedentary control group (CON) (n=10)
Gender (Woman n %)	4 (40%)	8 (80%)	4 (40%)
Age (Mean ± SD)	50.6 ± 6.1	52.6 ± 6.1	52 ± 5.9
Race/Ethnicity (n %)			
Non-Hispanic White	5 (50%)	4 (40%)	8 (80%)
Hispanic	1 (10%)	3 (30%)	0 (0%)
Black	2 (20%)	1 (10%)	1(10%)
Asian	3 (30%)	2 (20%)	1 (10%)
Pacific islander	1(10%)	0 (0%)	0 (0%)
Other	1 (10%)	1 (10%)	0 (0%)
Education some college (n %)	9 (90%)	8 (80%)	10 (100%)
Alcohol (#drinks/week)	1.6 ± 1.8	0.8 ± 1.9	0.7 ± 1.6
BMI (Mean ± SD)	23.4 ± 3.5	27.1 ± 2.5	27.1 ± 3.1

Abbreviations: BMI, body mass index (calculated as weight in kilograms divided by height in meters squared).
Race/Ethnicity: Multi-ethnic participants were allowed to respond more than one race/ethnicity category.
