# Supplementary material for: Oxylipin Dynamics Following A Single Bout of Yoga Exercise: A Pilot Randomized Controlled Trial Secondary Analysis
Source: J Integr Complement Med. 2024 Sep 16;30(9):897–901. doi: 10.1089/jicm.2024.0233 (PMC11807855; doi:10.1089/jicm.2024.0233)
Supplement: Supplementary Table S5 [file jicm.2024.0233_suppl_tables5.pdf]

S5 Table. Descriptive statistics for netAUC between groups for each lipid mediator.

Oxylipin family	Lipid mediator	Group[#]	N (netAUC)	Mean (ng.hr/mL)	Std Dev	Lower 95% CI for mean	Upper 95% CI for mean	Effect size[&]
Docosahexaenoic acid (DHA)- and Omega 3-derived oxylipins	RvD1	HY	9	-1.4	4.6	-5.0	2.1	0.7
		MY	10	-0.3	2.2	-1.8	1.2	0.6
		CON	9	1.1	2.4	-0.7	2.9	
	RvD2	HY	9	2.5	4.3	-0.8	5.8	0.3
		MY	7	2.2	3.3	-0.9	5.2	0.4
		CON	9	3.9	4.5	0.4	7.3	
	RvD3	HY	5	4.4	2.2	1.7	7.1	0.1
		MY	7	10	26	-14	34	0.3
		CON	6	5.0	9.9	-5.4	15	
	RvD5	HY	9	-0.4	4.6	-3.9	3.1	0.2
		MY	8	-2.0	7.2	-8.0	4.0	0.4
		CON	9	0.4	4.6	-3.1	3.9	
Eicosapentaenoic acid (EPA)- and Omega-3-derived oxylipins	RvE1	HY	8	-7.1	12	-17	3.2	0.8
		MY	7	4.4	17	-11	20	0.1
		CON	9	3.5	13	-6.9	14	
	18-HEPE	HY	7	2.5	11	-8.0	13	0.0
		MY	8	1.8	6.2	-3.4	7.0	0.0
		CON	9	2.1	9.4	-5.2	9.4	
	EPA	HY	8	0.6	8.3	-6.3	7.5	0.4
		MY	9	3.5	11	-5.0	12	0.6
		CON	7	-2.5	7.7	-9.6	4.6	
Arachidonic acid (AA)- and Omega-6-derived oxylipins	LXB4	HY	6	23	34	-12	58	1.1
		MY	9	4.2	25	-15	23	0.4
		CON	10	-4.7	16	-16	6.7	
	LXA4	HY	7	4.8	16	-10	20	0.3
		MY	9	3.4	17	-9.7	16	0.2
		CON	10	0.24	15	-10	11	
	AA	HY	9	-3.3	16	-16	9.0	0.4
		MY	9	8.4	21	-7.8	25	0.2
		CON	8	4.2	18	-11	19	
	6-keto-PGF1α	HY	7	2.6	8.4	-5.2	10	0.9
		MY	9	0.8	9.7	-6.6	8.3	0.7
		CON	9	-7.8	14	-19	3.0	
	TxB2	HY	8	3.1	19	-13	19	0.4
		MY	8	4.9	9.0	-2.6	12	0.6
		CON	9	-4.3	22	-21	13	
	PGE2	HY	9	1.9	13	-7.7	12	0.1
		MY	7	0.6	11	-9.6	11	0.2
		CON	10	3.1	13	-6.0	12	
	PGD2	HY	8	15	18	-0.1	30	0.4
		MY	6	-2.1	10	-13	8.6	0.9
		CON	9	8.3	13	-1.6	18	
	12S-HHTrE	HY	8	-5.0	22	-24	14	0.7
		MY	8	0.5	16	-13	14	0.5
		CON	8	8.4	14	-3.2	20	
	15S-HETE	HY	7	2.9	11	-7.7	13	0.0
		MY	6	8.0	5.6	2.2	14	0.4
		CON	10	2.3	23	-14	19	
	12S-HETE	HY	7	4.0	20	-14	22	0.4
		MY	7	3.5	17	-12	19	0.5
		CON	7	12	17	-3.7	28	

[#]High-intensity yoga exercise group (HY), moderate-intensity yoga exercise group (MY), and control group (CON).
[&]Cohen's d effect size: |HY-CON|/Average(SDcon+SDHY)/2; |MY-CON|/Average.
